# Machine Learning Techniques for Sensor-Based Human Activity Recognition with Data Heterogeneity—A Review

**DOI:** 10.3390/s24247975

**Published:** 2024-12-13

**Authors:** Xiaozhou Ye, Kouichi Sakurai, Nirmal-Kumar C. Nair, Kevin I-Kai Wang

**Affiliations:** 1 Department of Electrical, Computer, and Software Engineering, The University of Auckland, Auckland 1010, New Zealand; xye685@aucklanduni.ac.nz (X.Y.); n.nair@auckland.ac.nz (N.-K.C.N.); 2 Department of Informatics, Kyushu University, Fukuoka 819-0395, Japan; sakurai@inf.kyushu-u.ac.jp

**Keywords:** human activity recognition, data heterogeneity, data out of distribution, time-series classification

## Abstract

Sensor-based Human Activity Recognition (HAR) is crucial in ubiquitous computing, analyzing behaviors through multi-dimensional observations. Despite research progress, HAR confronts challenges, particularly in data distribution assumptions. Most studies assume uniform data distributions across datasets, contrasting with the varied nature of practical sensor data in human activities. Addressing data heterogeneity issues can improve performance, reduce computational costs, and aid in developing personalized, adaptive models with fewer annotated data. This review investigates how machine learning addresses data heterogeneity in HAR by categorizing data heterogeneity types, applying corresponding suitable machine learning methods, summarizing available datasets, and discussing future challenges.

## 1. Introduction

Human Activity Recognition (HAR) refers to the automatic detection and classification of human actions or behaviors using various data sources. HAR plays a pivotal role in ubiquitous computing by enabling intelligent services that can enhance daily life, improve health outcomes, and ensure safety. Some of the key applications of HAR include the following:

- **Ambient Assisted Living**: Supporting independent living for the elderly and individuals with disabilities by monitoring daily activities [1].
- **Fitness and Sports**: Enhancing performance tracking and providing personalized feedback for athletes and fitness enthusiasts [2].
- **Rehabilitation**: Assisting in the recovery process by monitoring and analyzing patient movements [3].
- **Security Surveillance**: Enhancing security systems by detecting suspicious or unusual activities [4].
- **Health Monitoring**: Continuously tracking health-related activities to prevent and manage medical conditions [5].

HAR methodologies are broadly classified into two categories: sensor-based and camera-based approaches.

Sensor-based HAR leverages data from embedded and wearable devices, which have seen widespread adoption due to their portability and ability to collect continuous data. The proliferation of these devices has made sensor-based HAR highly popular, finding applications across various domains, as mentioned above.

Camera-based HAR, on the other hand, utilizes cameras or video data to recognize human activities. While this approach can capture rich contextual information and detailed movements, it also presents challenges such as performance degradation under low-light conditions, obstructed camera viewpoints, and significant privacy concerns.

Given these considerations, this review primarily focuses on sensor-based HAR, particularly addressing the challenges associated with data heterogeneity. Data heterogeneity in sensor-based HAR encompasses variations in sensor types, data formats, and environmental conditions, which can significantly impact the accuracy and reliability of activity recognition systems.

**Figure 1:**
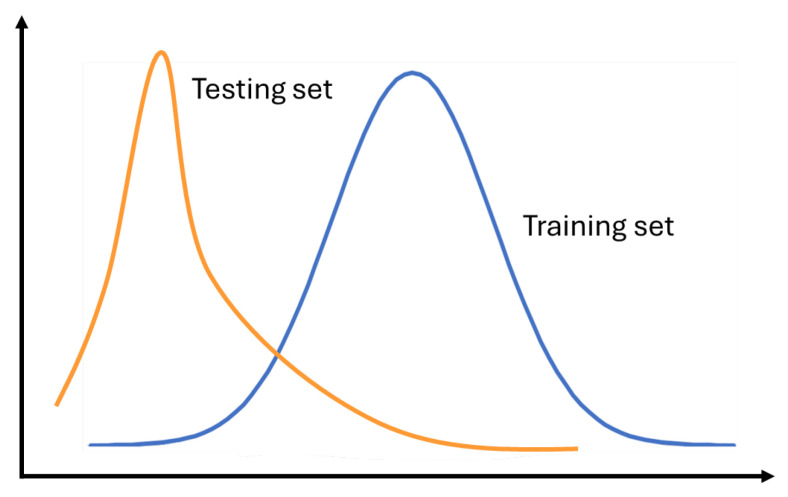
Non-uniform distributions of sensor-based HAR data.

Currently, most state-of-the-art sensor-based HAR machine learning algorithms assume that the test and training samples satisfy the hypothesis of IID (Independent and Identically Distributed) data [6] to ensure the generalization ability of the sensor-based HAR model. However, the practical situation often deviates from this ideal scenario encountered in experimental environments, as shown in Figure 1, leading to a dramatic decline in model performance. For instance, different individuals exhibit varying physiological characteristics, such as age, weight, and height, resulting in distinct activity data distributions. This variation in data distributions is commonly referred to as data heterogeneity [7].

Data heterogeneity frequently arises in embedded and IoT sensors, where datasets collected from diverse devices, users, and environments exhibit non-uniform distributions. This poses a significant challenge to conventional HAR methods, as their performance typically depends on consistent and uniform data assumptions. Although data heterogeneity is a common issue across the AI community, its manifestation in sensor-based HAR is uniquely influenced by the specific application context [8]. Addressing data heterogeneity can greatly enhance model performance; reduce computational costs; and facilitate the development of personalized, adaptive, and robust HAR models with minimal annotation effort [9].

Given the complexity and variability of HAR data, traditional rule-based or statistical methods are insufficient for capturing the intricate relationships in heterogeneous datasets. This has led to the increasing adoption of machine learning-based approaches, which offer several advantages for sensor-based HAR:

- **Learning Complex Patterns:** Machine learning algorithms can automatically identify and learn intricate patterns and dependencies in large-scale sensor data, enabling superior performance in recognizing diverse human activities.
- **Adaptability to Non-Stationary Data:** Modern machine learning models, particularly deep learning techniques, can adapt to non-stationary and heterogeneous data, making them well-suited for real-world scenarios.
- **Generalization Across Scenarios:** Through transfer learning, domain adaptation, and other advanced techniques, machine learning methods can generalize effectively across users and environments, addressing the challenges of data heterogeneity.
- **Reduced Manual Effort:** Machine learning approaches minimize the need for hand-crafted features and domain-specific expertise, streamlining the model development process.
- **Support for Personalization:** With techniques like meta-learning and federated learning, machine learning methods enable the development of personalized HAR models that cater to individual users’ unique characteristics and preferences.

Therefore, machine learning has become a fundamental component of sensor-based HAR research, driving innovations aimed at tackling data heterogeneity.

In this review, we seek to answer the following research questions:

1. What are the types of data heterogeneity in sensor-based HAR?
2. What are the state-of-the-art machine learning techniques developed for addressing the heterogeneity of sensor-based HAR data?

This review analyzes recent advancements in the field, providing insights into the suitability of various machine learning approaches for different types of data heterogeneity. Furthermore, it identifies gaps that warrant further exploration. To the best of our knowledge, this is the first review that specifically focuses on data heterogeneity issues in sensor-based HAR applications.

In order to keep a clear focus, we applied some constraints to our survey. This review focuses on the sensor-based HAR data heterogeneity issues, and camera-based HAR is not considered in this study unless it is used in conjunction with sensor-based approaches. Also, this review specifically focuses on sensor-based HAR for general physical and daily activities such as walking, jumping, taking a shower, washing dishes, etc. Application-specific activities such as fall detection or human disease/ill responses are not covered in this review.

The remainder of this paper is structured as follows. Section 2 summarizes and categorizes the different types of data heterogeneity in sensor-based HAR, followed by a background introduction to the typical machine learning paradigms. Section 3, Section 4, Section 5, Section 6 and Section 7 review different machine learning paradigms in different types of sensor-based HAR data heterogeneity, namely, data modality heterogeneity, streaming data heterogeneity, subject data heterogeneity, and spatial data heterogeneity, as well as a general framework for multiple heterogeneities. Section 8 introduces the available public datasets for various types of data heterogeneity in sensor-based HAR. Finally, future directions and conclusions are presented in Section 9.

## 2. Data Heterogeneities and Machine Learning Paradigms in Sensor-Based HAR

In this section, we introduce the types of data heterogeneity and the related machine learning paradigms used in this paper. To solve the issue of data heterogeneity in sensor-based HAR, several machine learning paradigms have been applied by the research community. The focus of this review is on how these paradigms could contribute to solving the issue of sensor-based HAR data heterogeneity.

### 2.1. Sensor-Based HAR with Data Heterogeneities

Sensor-based HAR aims to automatically identify and classify human activities using data collected from various sensors. It provides valuable insights into how humans move, behave, and interact with their environment. We use the following definition of sensor-based HAR as described in previous work [10,11,12]:

Sensor-based human activity recognition (HAR) refers to the process of inferring human actions or behaviors from raw sensor data, typically collected using embedded, wearable, or environmental sensors. These sensors capture various modalities, such as acceleration, gyroscope readings, heart rate, and environmental parameters, which are then analyzed to determine the performed activity. By utilizing data from sensors such as accelerometers, gyroscopes, and magnetometers, sensor-based HAR systems can recognize a wide range of activities, including walking, running, sitting, or even more complex actions such as cooking or exercising. The ultimate goal is to create intelligent systems that can understand and adapt to human behavior in diverse real-world applications.

Here, we introduce the sensor-based activity categories and the sensor-based activity recognition data heterogeneity categories. We refer to previous work on sensor-based HAR taxonomy [6] and present the four categories of sensor-based activity.

Atomic activity [13] refers to a basic or simple activity that is a component of a more complex activity. An atomic activity is a fundamental action that often lasts for a very short time. It is normally treated as the minimum component of human activity. For example, crossing arms forward, waist bends, kicking, and knee bending can be considered atomic activities. Daily living activities refer to the repeated activities that people perform on a daily basis. They often involve performing the same action in the same way over and over again. For example, brushing teeth involves the repeated movement of a toothbrush in a specific pattern. Sports fitness activities refer to the physical movements and exercises performed during sports and fitness activities. Repetitive activities are often a key component of sports and fitness training, as they help to build strength, endurance, and muscle memory. These activities may include running, cycling, weightlifting, and other exercises that are designed to improve physical fitness. A composite activity [6] can be defined as a sequence of sub-actions and have higher-level semantics. Composite activity recognition is a more challenging task than recognizing simple activities because it requires not only the detection human body movements but also consideration of contextual information about the surrounding environment. For example, “making coffee” can be represented as a sequence of simple activities that happens in a kitchen environment. Table 1 shows the four categories of sensor-based activity with the corresponding activities.

**Figure 2:**
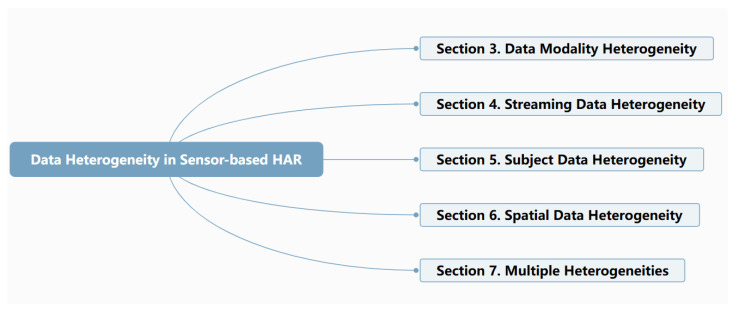
Sensor-based HAR data heterogeneity categories.

Data heterogeneity is a commonly occurring challenge in IoT data, where collected datasets have non-uniform distributions [8]. Considering sensor-based HAR applications, there are various sources of data heterogeneity. First, data can be generated by a variety of sensor devices, which is called data modality heterogeneity. Different sensor types, platforms, manufacturers, and modalities may cause the different data formats and distribution. Secondly, dynamic streaming data patterns may change against time if the properties vary under a certain influence, which is called streaming data heterogeneity. For example, the walking pattern of the same person may be affected by health and physical status. Thirdly, behavior may differ between people, which is called subject data heterogeneity. For instance, some people may make coffee in the evening, while others may drink coffee in the morning. Fourthly, different sensor networks in different body positions or different environmental layouts in smart homes may lead to different data distributions, which is called spatial data heterogeneity. Finally, the mixed sensor-based HAR data heterogeneity may occur, requiring a general framework to handle the issue. Figure 2 shows the five sensor-based HAR data heterogeneity categories.

### 2.2. Machine Learning Paradigms

**Figure 3:**
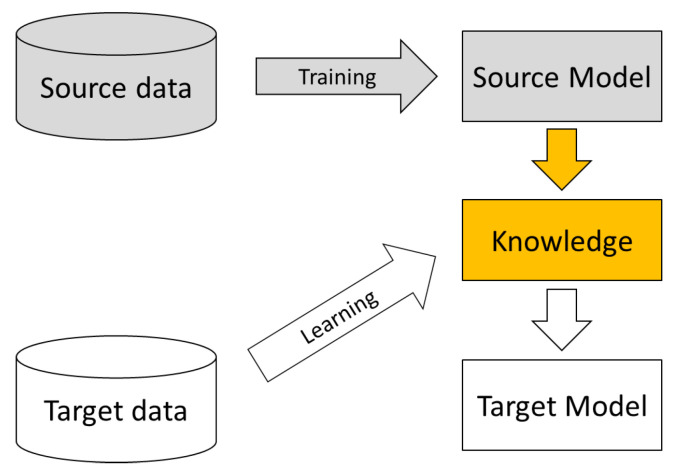
Transfer learning workflow.

For all the abovementioned different types of sensor-based HAR data heterogeneity issues, transfer learning is the most common machine learning paradigm that can be applied. Transfer learning [14,15] is a machine learning paradigm that captures knowledge from one predefined problem and applies it to a different problem. This paradigm covers a wide range of scenarios, and all transfer learning techniques emphasize the learning direction from the source domain(s) to a target domain and assume the knowledge in the source domain(s) could be helpful in the target domain, as shown in Figure 3. Transfer learning is particularly suitable for dealing with the sensor-based HAR data heterogeneity problem and is the dominant method. In the sensor-based HAR field, typically, an existing dataset(s) with labeled data can be seen as source the domain(s). Then, a new dataset that the system has never seen before, such as a new activity, a new user, a new environment, or a new device, is considered as the target domain.

In the data modality heterogeneity field, multi-view learning is particularly suitable because each sensor modality can be considered as a distinct view to provide knowledge. Multi-view learning [16] focuses on data representation via multiple distinct views to improve the generalization performance. This learning paradigm is based on the idea that two heads are better than one, integrating the advantages of different views. It aims to learn only a single task with all the views, as opposed to multi-task learning, which learns for many different tasks.

In the streaming data heterogeneity field, continual learning, zero-shot learning, and few-shot learning are the three most common machine learning paradigms. Continual learning [17], lifelong learning, or incremental learning can process the flow of information over time; update the model continuously; and retain, integrate, and optimize existing knowledge while absorbing new knowledge from emerging samples. This paradigm focuses more on multiple sequential domains and tasks from a temporal dimension and implicitly copes with the different distributions and data heterogeneity across domains.

Zero-shot learning [18] is another subfield of machine learning that focuses on classifying emerging classes. Typically, there are some labeled training samples, and these data classes are called seen classes. However, several unlabeled testing samples of different classes are referred to as the unseen classes in the label space. This paradigm aims to identify these unseen classes. In zero-shot learning, the source-domain feature space of training instances is the same as the target-domain feature space of testing instances. However, the source-task label space (the seen class set) is different from the target-task label space (the unseen class set). Zero-shot learning is suitable for the scenario of new activities.

Few-shot learning [19] aims to train a model using only a few training examples based on prior knowledge of several similar tasks. One particular example of this is ‘K-shot, N-way’ classification, which implies a trained classifier that needs to classify N new classes using only K examples of each. When adding the restricted condition of only one instance for each class in few-shot learning, it becomes so-called one-shot learning. In sensor-based HAR, scarce labeled data in the target domain are very common.

In the subject data heterogeneity field, federated learning, multi-task learning, and ensemble learning are the three most common machine learning paradigms. Federated learning [20] was originally designed for distributed machine learning, emphasizing data security and privacy. Because of its good characteristic of sharing information across all users when performing coordinated training, it has attracted increasing attention to solve the issue of data heterogeneity in sensor-based HAR with multiple users. Normally, the data are heterogeneous across various users, and this paradigm can be utilized to improve each user’s performance in a safe way. When the sensor-based HAR challenge is multi-user data heterogeneity, federated learning is worth a try.

Multi-task learning [21] pays attention to the collaborative and distributed learning of multiple tasks with shared models. The goal is to improve the performance of all the models with heterogeneous data. Like transfer learning, multi-task learning aims to improve the performance of learners via knowledge transfer. However, transfer learning focuses on the one-way transfer of knowledge from the source domain(s) to the target domain(s). Multi-task learning focuses on knowledge transfer across tasks and mainly takes advantage of the interconnections between tasks. Multi-task learning is especially useful in scenarios of data heterogeneity in sensor-based HAR with multiple using considering multiple different tasks.

Ensemble learning [22] involves combining several base classifiers to improve the final model’s generalization and robustness. Ensemble learning is based on the idea of achieving better performance when there is significant diversity among the base classifiers. Each base classifier contains some knowledge, and multiple base classifiers can provide more diverse and comprehensive knowledge. Therefore, the weights of different knowledge sources can be adjusted to flexibly build a model to deal with sensor-based HAR data heterogeneity issues.

In the spatial data heterogeneity field, multi-view learning can also be applied because it can synchronously handle environmental layout heterogeneity across multiple houses. Moreover, domain generalization can be applied to handle scenarios of multiple heterogeneities in sensor-based HAR. Domain generalization [23] is a particular type of transfer learning that aims to learn a model that can generalize to a target domain with one or several different but related source domain(s). Compared to common transfer learning training, in which data from both the source and target domains can be accessed, domain generalization can only access several pieces of source-domain data for training, and target-domain data are not accessible. Therefore, domain generalization aims to tackle more challenging and practical scenarios.

**Figure 4:**
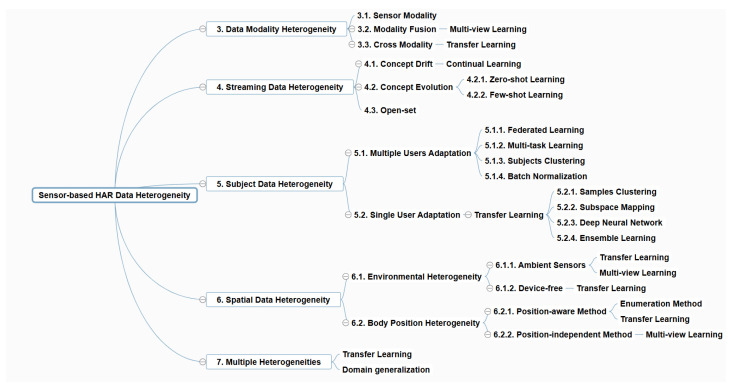
Overall machine learning technique framework.

In the following sections, we discuss different machine learning techniques applied to the five types of HAR data heterogeneity, which are summarized and illustrated in Figure 4.

## 3. Data Modality Heterogeneity

We begin by discussing data modality heterogeneity in sensor-based HAR. Various types of data modalities are employed in different scenarios to implement sensor-based HAR, as summarized in Table 2. The performance of such systems is highly dependent on the choice of sensor modalities used as data sources. Machine learning techniques for addressing data modality heterogeneity are mainly partitioned into modality fusion and cross-modality methods. Modality fusion aims to combine and integrate multiple sensor modalities into a unified form using multi-view learning. For example, IMU sensor-based HAR can be combined with camera-based HAR to provide broader views and compensate for the limitations of IMU sensors alone. Cross-modality methods focus on transforming data from one modality to another to achieve certain goals under transfer learning paradigms. For instance, knowledge from camera-based HAR can be transferred to sensor-based HAR to enhance its performance.

### 3.1. Sensor Modality

Sensor modalities are classified into three types: inertial measurement unit sensors, ambient sensors, and device-free sensors [24,25,26].

IMU sensors measure the angular rate; force; and, sometimes, magnetic fields. They are portable and can be deployed at different body positions, such as the waist, ankle, arm, leg, back, and chest, to directly capture data on body context. IMU sensors can also be embedded in smartphone carried in pockets or held in hands to collect data. They are very common in HAR research and have broad application scenarios such as daily activity recognition [27], sports activity recognition [28], and working activity recognition [29].

Ambient sensors are typically deployed in the environment at fixed positions to capture interaction information between humans and their surroundings. In HAR scenarios, they are essential devices in smart homes (or related applications) and can be embedded with additional position information.

Device-free sensors represent a new trend in wireless sensing research for HAR. Wireless signals can not only be utilized as communication tools to exchange data but also have the ability to reflect differences in various human behaviors and sense variations in the surrounding environment. This type of sensor normally has a transmitter and receiver to send and receive electromagnetic waves. Different human activities lead to different reflected signal patterns. Device-free sensors can usually be deployed on the walls or ceilings of a room.

### 3.2. Modality Fusion

The purpose of modality fusion is to integrate various sensor modalities to improve the performance of sensor-based HAR systems. The mainstream learning paradigm in modality fusion is multi-view learning, which treats each sensor modality as a separate view. Multiple views synchronously observe the same activity and unite to accomplish the common activity classification task. There are mainly two types of modality fusion: data-level/feature-level fusion and classifier-level fusion. The human activities researched in modality fusion include activities such as going up stairs, going down stairs, walking, biking, standing, pressing buttons, plugging in cables, rotating chairs, using hammers, clapping, crossing arms, bowling, tennis serves, and baseball swings.

Data-level/feature-level fusion extracts features from each sensor and combines them to train a single classification model. The most common approach in feature-level fusion is aggregation, which concatenates the extracted features or certain raw data from all sensors. Ehatisham et al. [30] focused on feature-level data fusion from two modalities: RGB/depth video camera and inertial body sensors. They extracted features separately, then concatenated the two feature vectors. These features include densely extracted histograms of oriented gradient features from the camera and statistical time-series characteristics from wearable sensor data. Li et al. [4] combined a Kinect depth camera and wearable sensors as a complementary fusion to improve performance. The depth data features and wearable sensor data are joined in a data-level fusion. Data contraction is realized by feature selection with metaheuristic methods. Finally, incremental learning is applied via the Hoeffding tree and the swarm decision table.

Inspired by the design of the non-local block in self-attention deep networks, which is used to compute relations between different space-time locations of the same instance, Byvshev et al. [31] adapted the non-local block for heterogeneous data fusion of video and smart-glove data. There are two main modifications: (1) changing from self-attention to cross-modal attention via two multi-modal inputs replacing the input in the original design and (2) adjusting the non-local operation in a way that does not require the matching of spatio-temporal dimensions for data fusion.

In contrast to most work in this field with relatively big differences in modalities, such as ambient sensors and video, Stisen et al. [32] conducted systematic research on the heterogeneity of 36 smartphones, smartwatches, and tablets with various device models from four manufacturers. Data heterogeneity mainly originates from sensor biases (poor sensor quality or accidental device damage), sampling rate heterogeneity (different device factory settings), and sampling rate instability (varying I/O loads affecting the actual sampling rate). To mitigate the effects caused by heterogeneities, the kNN clustering method is applied to group similar devices; then, a classifier is trained based on the devices’ sensor data in each cluster.

Classifier-level fusion aims to combine classifiers trained on each sensor modality to build a more robust model. This fusion strategy overcomes the drawback of feature-level fusion of feature compatibility issues regarding heterogeneous sampling frequencies and configuration parameters. Garcia-Ceja et al. [33] proposed a stacking ensemble-based multi-view learning method for modality fusion. Unlike traditional multi-view learning via aggregation, which mixes the feature spaces from multiple views, this method builds individual models for each view and combines them using stacked generalization. The highlight of this paper is the comparison of the audio view, accelerometer view, aggregation view, and multi-view stacking, with results showing that multi-view stacking achieves the best performance.

Using the same stacking ensemble technique as Garcia-Ceja et al. [33], Chung et al. [34] extended the meta-learner to include a voting strategy and compared it with the stacking method. They also compared three machine learning models—Random Forest (RF), k-Nearest Neighbors (kNN), and Support Vector Machine (SVM)—in stacking ensembles. Xue et al. [35] proposed a deep learning architecture for unified multi-modality fusion that considers the different sensors’ data quality for information-weighted adjustment and the correlations among various sensors. The sensor-representation module is a Convolutional Neural Network (CNN) structure that extracts low-dimensional features of the same size, even with non-uniform heterogeneous input. The weighted-combination module is inspired by the attention mechanism, which estimates the quality of each sensor’s data and combines the information in a weighted manner. The cross-sensor module captures the correlations among the various sensor information to learn a more robust model via an averaging operation.

As shown in Table 3, data-level/feature-level fusion follows a fine-grained fusion approach, considering all the features/data in different modalities together. After the learning process, the weighted contributions of the features/data for the final task can be obtained. In this way, only one classifier is required, which saves training time and computational resources. However, unifying different modality features/data into the same format is an extra step, and feature/data compatibility issues need to be carefully addressed.

The classifier-level fusion approach is a coarse-grained fusion method that focuses on the co-decision of multiple classifiers. It is convenient because there is no need to consider the compatibility issues of different modalities, and each modality can be trained based on its own common method. However, the different classifiers may have biases, and dealing with inconsistent results across classifiers is another problem. Regarding activity categories, data-level/feature-level fusion focuses on atomic activity, daily living activity, and sports fitness activity, while classifier-level fusion considers daily living activity.

### 3.3. Cross-Modality Methods

Cross-modality methods aim to transfer knowledge from one modality to another to assist in achieving goals with different data modalities. The main learning paradigm in cross-modality sensor-based HAR is transfer learning because of directional knowledge transformation, the goal of which is to transfer useful information from the source modality to the target modality. Cross-modality knowledge transformation aims to build a bridge and find the latent relationships between different data modalities to take advantage of knowledge from the source domain. There are four main branches in sensor-based HAR cross-modality approaches: deep neural network fine tuning, multi-view transfer learning, knowledge distillation, and generative network methods.

Xing et al. [36] proposed the deep learning fine-tuning method, which can transfer knowledge across vision, audio, and IMU sensors. For knowledge transfer, source-domain data are fed into a pre-trained model to calculate the activation values in an intermediate layer as a cross-domain shared feature vector. Then, target domain-data are used to train a network to map the shared feature vector to a unified feature space across multiple domains. For task transfer, if the tasks are the same between the source and target domains, the source-domain model’s higher layers for classification can be reused directly in the target-domain model. If the tasks are different, the target model’s low and intermediate layers are frozen, and the target model’s higher layers are re-trained using limited target-domain data.

Feuz and Cook [37] introduced a multi-view transfer learning algorithm applicable with or without labeled data in the target domain. Ambient sensors, smartphone/wearable sensors, and video cameras use transfer learning to act as colleagues. In scenarios where a small amount of labeled data is available in each view, a separate weak classifier is trained for each view; then, all classifiers select the unlabeled samples to add to the labeled set based on a confidence score. In scenarios with unlabeled target-domain data, subspace learning is utilized to project the source and target domains onto a latent feature space via Principal Component Analysis (PCA) dimensionality reduction. After aligning the subspaces between views via Procrustes analysis, projected data from the source view are used to train a classifier that tests projected data from the target view for data annotation. Finally, a classifier is trained using labeled data in the target view.

Kong et al. [38] presented a knowledge distillation method for transferring knowledge from a wearable sensor modality to a video camera modality. Knowledge distillation allows the student network to capture the information provided by the ground-truth labels and the finer structure learned by the teacher network [39]. In the first step of generating the teacher model, multi-view learning is applied to multiple wearable sensors to train a classifier for each sensor; then, a weighted adaptive method is used to combine the classifiers according to their feature representations. In the second step, the teacher model’s knowledge is transferred to the student model by training with classification loss and distillation loss.

**Figure 5:**
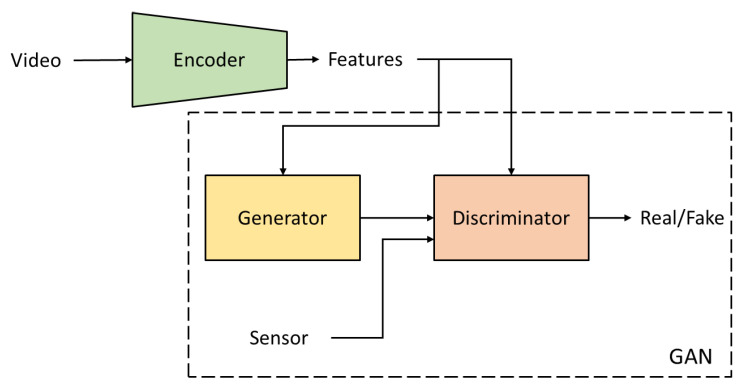
The framework of the conditional Generative Adversarial Network (cGAN) for generating synthetic sensor data from video features.

Zhang et al. [40] worked on deep generative networks for transferring video data to synthetic accelerometer data. They proposed two algorithms: a conditional generative adversarial network (cGAN) and conditional variational autoencoder-conditional GAN (cVAE-cGAN). For cGAN, a video encoder is used to compress the video clip into a feature vector. Then, a GAN models the conditional distribution of sensor data based on the video feature vector, as shown in Figure 5. cVAE-cGAN is an extended version of cGAN. A cVAE uses the information from the video to learn a conditional distribution of the sensor data based on the idea that prior knowledge from cVAE will improve the generative capability of the GAN.

As shown in Table 4, among the cross-modality approaches, multi-view transfer learning is the only method that can be applied in scenarios without labeled target-domain data. It considers various classifiers’ results and provides comprehensive views for the target domain’s generation of pseudo-labels, leveraging the wisdom of the masses. However, more training resources are required. In contrast, fine tuning is a more computationally resource-friendly method and does not require training of the model from scratch. The drawback of fine tuning is that performance may drop significantly if the new task is very different from the original task. Knowledge distillation trains the distilled model on the combination of the original model’s predictions and the ground-truth labels, which may improve generalization to new data compared to the original model. However, this type of model compression technique may not capture all the information and nuances present in the original model, potentially resulting in lower performance on certain tasks. The generative network can produce a large amount of synthetic target-domain modality data, which can be useful for data augmentation. However, the generated data may not capture all the nuances and details present in real data, possibly resulting in lower quality or unrealistic outputs. The corresponding human activity categories in each approach are also listed in Table 4. Fine tuning and generative networks focus on daily living activity and sports fitness activity. The knowledge distillation approach aims to handle daily living activity and atomic activity, while multi-view transfer learning focuses on composite activity.

## 4. Streaming Data Heterogeneity

Sensor-based HAR often requires continuous data streaming. Typically, sensor data is a continuous and infinite data stream with a high sample rate and variable data distribution. However, many researchers assume the data distribution remains the same over time to simplify the problem. Dynamic changes in activities over time are an inherent and natural characteristic of the sensor data stream. Variation in existing activities or the emergence of new activities occurs in evolving activity data streams [11]. For example, people’s activities in the morning may have different data distributions than same activities at night because of muscular fatigue. As for the instance of the emergence of new activities, people may perform new activities such as learning new outdoor activities. There are three main categories of streaming data heterogeneity, namely concept drift, concept evolution, and open-set issues.

### 4.1. Concept Drift

Concept drift [41] is a data heterogeneity issue in the temporal dimension that refers to distribution change over time. In the sensor-based HAR concept drift scenario, historical data are typically considered the source domain, an emerging data are considered the target domain. Continual learning is the mainstream learning paradigm in concept drift because it is typically applied to unlimited streaming data. Various methods have been proposed that focus on identifying when concept drift occurs and how to adjust the model to adapt to concept drift.

Roggen et al. [42] presented an adaptive continual learning framework. In comparison with typical supervised HAR models, this method has three additional steps for handling concept drift: self-monitoring, adaptation strategies, and exploitation of available feedback. Self-monitoring discovers relevant changes in the system’s performance for the identification of the possible concept drift point. Adaptation strategies adjust the parameters of the activity recognition model for acceptable performance after concept drift happens. Available feedback includes the user, the activity-aware application, and external systems guiding model adaptation. Because of the general framework design, various machine learning techniques can replace the components in the adaptive activity recognition chain framework.

Abdallah et al. [43] introduced a two-phase method (i.e., online and offline) to handle data streams. In the offline phase, a cluster dictionary is generated that includes a set of clusters with their corresponding sub-clusters. Sub-clusters are the different patterns for each cluster. Then, an ensemble classifier is generated based on several hybrid similarity measure approaches for prediction. This classifier deploys an ensemble of four measures to assess the similarity of new data related to the learned cluster dictionary. The online phase involves activity recognition and adaptation components. Upon the arrival of the new data stream, if each measure chooses a different cluster, it is highly possible that concept drift has happened, and the data are required to be annotated. Then, the adaptation component updates the sub-cluster inside the corresponding cluster based on the newly labeled data to adjust the similarity distance of the four measures to solve concept drift.

Lima et al. [44] presented a continual learning classification algorithm based on symbolic data generated by a discretization process using SAX algorithms [45]. First, each chunk is a time window that goes through a discretization process, and the data are transformed into symbols. Then, these symbols in each chunk are represented via histograms based on their frequency distributions. This generated dictionary of frequency distributions is the feature of the corresponding activity class. In the activity recognition step, the algorithm compares the frequency distributions between the histograms of new data and the dictionary of histograms in each class. If there are changes in the frequency distribution of histograms, concept drift is considered to have occurred. In the adaptation step, to handle concept drift, all existing histograms are retrieved to add some novel histograms that never appeared before, delete some histograms that do not occur any more, and keep the histograms that are still effective after concept drift.

Meng et al. [46] proposed a knowledge-based continual learning method that considers activity recognition and abnormal behavior detection together. Here, abnormal behavior is viewed as concept drift that has a different data distribution compared to normal behavior. The unique point of this paper is the design of a dynamic daily habit component that aims to learn the habits of elderly from their daily activities via the construction of a two-layer tree architecture. The nodes in the first layer refer to the activity classes, and the probability of the user performing each activity in a time period is calculated in the second layer. By doing so, the dynamic daily habit component can not only find the statistical patterns of users’ daily habits but also serve as a knowledge base assisting in concept drift detection for users’ daily lives. The purpose of this work is to identify concept drift; therefore, there is no model adaptation step to solve concept drift.

As Table 5 shows, the adaptive activity recognition framework and cluster ensemble classifier method need certain feedback and new labeled data when concept drift happens. In this way, it adds some extra manual effort but can rectify the model effectively to achieve a guaranteed performance. The cluster ensemble classifier method includes some high-confidence data based on its cluster similarity mechanism that reduce the degree of human feedback compared to the adaptive activity recognition framework. The adaptive activity recognition framework can be integrated into many machine learning techniques and components with better compatibility and scalability. The cluster ensemble classifier method and symbolic data discretization frequency distribution method essentially aim to find good feature representations that can be used for activity classification and different time period similarity measures. The symbolic data discretization frequency distribution method applies continuous time-series data compression techniques that reduce the algorithm’s computational requirements dramatically. However, it also leads to the loss of information, where a sensor value may be grouped into a different range of symbolic discretization that causes uncertainty issues affecting subsequent concept drift detection and adaptation. The generation of knowledge rules depends on expert experience and does not require any training data at all. However, a small variation in data distribution may violate the rules, and false detection may happen. The corresponding human activity categories in each approach are also listed in Table 5. The adaptive activity recognition framework aims to solve sports fitness activity, while the cluster + ensemble classifier has the extra category of daily living activity. The symbolic data discretization + frequency distribution method focuses on daily living activity, while the knowledge-based approach is designed for composite activity.

### 4.2. Concept Evolution

Concept evolution [47] means that new activities appear in a continuous data stream, and the new classes need to be incorporated into the existing classes. Typically, people may perform new behaviors that have never occurred in the past. Moreover, some rarely occurring activities or accidental events may happen unexpectedly, such as falling. Concept evolution aims to identify the concrete new activity instead of filtering and ignoring these new classes. Some methods in this area also belong to continual learning because of the characteristic of infinite streaming data. Some methods focus on zero-shot learning without new labeled data [18]. Some methods relax the strict limits of zero-shot learning and focus on few-shot learning and one-shot learning with limited new labeled data.

#### 4.2.1. Zero-Shot Learning

Zero-shot learning applied to concept evolution focuses on capturing the semantic relations between old concepts and new concepts without new labeled concepts [48]. Wang et al. [49] proposed a method for zero-shot learning via the learning of a non-linear compatibility function between feature space instances and semantic space prototypes. Here, prototype means a 0–1 vector; if instances of a class have an attribute, the corresponding digit in the class prototype is “1”. In the training phase, compatibility-based methods aim to learn a function measuring the degree of compatibility between a feature space instance and a semantic space prototype to build the similarity relationship between the two spaces. In the testing phase, the testing instance’s compatibility score is calculated with all unseen class prototypes, and the unseen class with the highest score is assigned to the instance.

Machot et al. [50] investigated off-site assistance and considered the semantic distance between different activity words in the natural language semantic view. In the training phase, the names of the specific activities are encoded in word vectors via Google Word2Vec representation. These word vectors become the training labels, which is different from the common method of encoding classification labels. Then, the sensor readings of the activities and the corresponding word vector labels are fed into a shallow neural network for model training. When concept evolution occurs, the new unseen sensor readings are sent to the neural network to obtain the output of a word vector. The word vector is applied to the nearest neighbor matching algorithm to find the most similar concept. This method takes advantage of the semantic natural language relationship to provide extra information for zero-shot learning.

In contrast common methods taking advantage of the semantic relationships between old and new concepts, reference Hu et al. [51] focused on the assumption that a new class label is independent of past labels. Some common time-series features are utilized in measuring the relation between old concepts and new concepts. An Axis-Aligned minimum Bounding Box (AABB) is introduced into the random forest (RF). A separating axis theorem based a splitting strategy is proposed with AABB, which enables the decision tree to be inserted with new nodes or split a leaf node without changing the original structure of the decision tree. From the geometry view, AABB is the box with the smallest surrounding space in which all the discrete points lie. To enable the insertion of a parent node, AABB is introduced to construct an incremental learning decision tree. It is used to find the intersection between an existing node and new data. Moreover, the Separating Axis Theorem is introduced to find an appropriate attribute and position to split the decision tree when concept evolution happens.

Hartmann et al. [52] proposed an interactive method that asks the user to annotate new data when new activities emerge. Then, the classification model is re-trained on the device with new activities, and the new model is loaded when a user decides to switch to the new recognition model.

#### 4.2.2. Few-Shot Learning

Compared to zero-shot learning, few-shot learning has loosens the restriction on new data. With limited labeled data, the model is able to adjust further to fit the new activity classes, and the performance of the system can be improved. Wijekoon et al. [53] used a neural network with an attention mechanism to integrate new concepts into an existing model when concept evolution happened. During the training step, the data are divided into two parts: a small number of labeled data called the support set and the rest of the labeled data called query instances. Then, a neural network is used to learn the common representative features for transforming both parts of the data into feature vectors. The cosine similarity metric is calculated for to measure the similarity between all query instances and support-set instance pairs. In addition, an attention mechanism is utilized to estimate the class distribution. The pair-mapping distance between the query instance and support-set instances that belong to the same activity class is minimized via neural network training. When concept evolution occurs, the distance of the new class to the support set is large. Then, the cosine similarity is calculated between the new data to the support set, and the attention weight is updated.

The technique presented in [54] integrates two advanced continual learning methods called Net2Net and Gradient Episodic Memory (GEM) to identify new concepts. When concept evolution occurs, Net2Net expands an already trained network by adding more neurons and layers to improve the learning ability to distinguish new concepts. The model is often extended from the last layer to fit new classes and from the second last layer to improve the learning capacity to discriminate a wider range of classes. GEM reduces the forgetting effect by balancing the performance of old and new classes and preventing the disastrous forgetting of old concepts by controlling gradient updates. GEM ensures that the loss from previous tasks does not escalate after each parameter adjustment to avoid forgetting.

Ye et al. [55] proposed a GAN-based method that preserves old concepts without storage of historical data. When new concepts occur, samples generated by the GAN combined with new data are used for classification. First, the GAN and a classifier, which is a deep neural network, are trained based on the current training data. When emerging data contain new concepts, the GAN generates samples for the previous activities with the learned latent structure. With the generated samples and the emerging data, the classifier is updated to recognize activities for both old and new concepts. The GAN is also updated to generate samples for the new activities. The above process repeats iteratively during the concept evolution process.

#### 4.2.3. Comparison of Methods

As shown in Table 6, zero-shot learning aims to learn new concepts without labeled data. The mainstream methods focus on mining the relations between new concepts and old concepts utilizing historical knowledge for emerging knowledge. However, if the new concepts have no or weak relations with old concepts, then the historical knowledge becomes less effective for the learning of new concepts. Few-shot learning requires a certain amount of labeled data for a new concept. The limited amount of labeled data guides the learning direction. However, if the new labeled data are not representative enough and only cover a subset of the complete concepts, then the concept evolution direction may be misunderstood, and the new concepts may not be learned properly. The corresponding human activity categories in each learning approach are also listed in Table 6. Both zero-shot learning and few-shot learning focus on daily living activity, sports fitness activity, and atomic activity. However, only zero-shot learning has been applied to composite activity.

### 4.3. Open-Set Recognition

Open-set recognition [56] is a new and trending research direction. It considers the practical scenario of incomplete knowledge of the world at training time, and unseen classes can be fed into the algorithm at testing time. The requirements of this type of approach are to accurately classify the seen classes and effectively reject unseen classes. Compared to concept evolution, open-set recognition does not need to identify specific unseen classes and only needs to treat the unseen classes as a whole part. Based on the idea of constructing a negative set to represent the unseen activity space [57], the open-set problem is simplified as “the seen classes plus one negative set class” classification problem. Moreover, a GAN deep learning model is proposed to learn to generate the negative set of synthetic data. After the training process, the discriminator part of the GAN is the classifier handling the open-set issue.

## 5. Subject Data Heterogeneity

Another commonly occurring sensor-based HAR data heterogeneity issue is caused by different subjects. For example, different people have different walking patterns considering variations in age, weight, and height. The relevant techniques can be considered mainly in two categories. One type of technique aims to improve multi-user model performance in general, whereas the other type aims to improve individual user model performance.

### 5.1. Multi-User Adaptation

**Figure 6:**
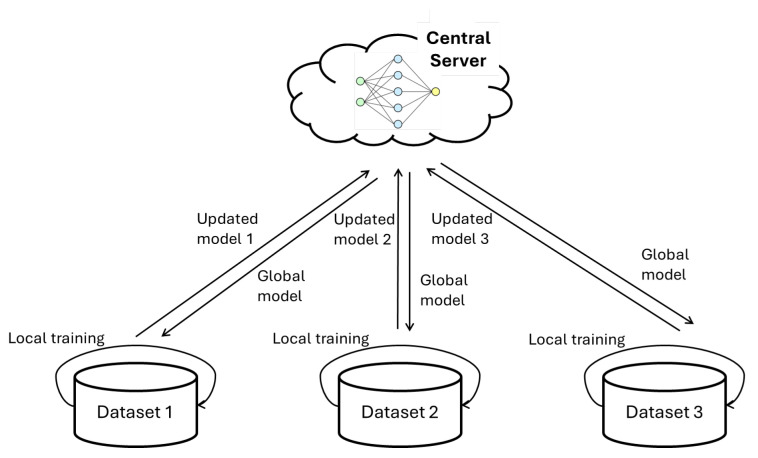
Federated learning workflow.

One of the most popular and noticeable approaches to multi-user sensor-based HAR multi-adaptation is federated learning. Federated learning benefits multiple subjects with data heterogeneity by integrating the subjects’ local models into a center model and sharing the center model with each subject for local model performance improvement [58]. This is an iterative process, and eventually, the knowledge from client models is shared through model parameters across different clients, as shown in Figure 6.

#### 5.1.1. Federated Learning

In the sensor-based HAR multi-user data heterogeneity scenario, federated learning focuses on learning invariant features across multiple users; these invariant features can also be efficiently used in activity classification. The essence of federated learning is knowledge sharing [59] among multiple users. Non-IID data introduce challenges such as higher communication overhead, imbalanced class distributions, and uneven updates to local models, all of which impact the convergence and performance of federated learning [60].

Some works have focused on the federated learning strategy to deal with the multi-user data heterogeneity problem. Sannara et al. [61] evaluated and compared the performance of three types of federated learning strategy, namely FedAvg, FedPer, and FedMa. FedAvg (Federated Averaging) aims to aggregate the local model updates from multiple clients by computing the average of the model parameters. FedPer (Federated Personalization) is an extension of FedAvg that takes into account the heterogeneity of the client data by weighting the local model updates based on the similarity between the local and global models. FedMa (Federated Moving Average) is another extension of FedAvg that uses a moving average to smooth out the fluctuations in the local model updates and reduce the noise in the global model. The result shows that FedAvg achieves the best generalization performance on clients. Presotto et al. [62] proposed a novel federated learning strategy that clusters similar users to form multiple groups to mitigate the side effect of different data distributions of users. The intuitive idea is that similar users share similar sensor data patterns, and if two client models share similar weights, then the corresponding users are likely to have similar patterns of activities and a small degree of data heterogeneity. Therefore, the weights of client models in the last several layers are used to calculate the cross-user similarity score.

Building upon this concept, Zhou et al. [63] enhanced this clustering strategy by incorporating social context attributes, such as gender, age, and weight, into the participant selection process. They proposed a hierarchical framework that utilizes social context clustering to form edge participant groups, ensuring that participants with similar contextual attributes are grouped together. This approach facilitates a more efficient and targeted federated learning process by dynamically identifying participants with high-quality, representative data. Each edge group independently performs local federated learning to generate group-specific models. These group models are then aggregated at a higher level to produce a more robust and generalized global model.

Chen et al. [64] proposed a CNN-based federated learning framework with an extra data distribution component to mitigate the data heterogeneity issue. The general framework is the same as the common federated learning framework. First, the center dataset is used to train the center model. Then, the center model is distributed to all the clients, and each of them can train their individual models on their own labeled data. After that, the client models are pushed to the cloud to iteratively update the center model. Lastly, each client re-trains its customized model by combining the newly trained cloud model. To further reduce the divergence between the center model and client models, a correlation alignment layer is added before the softmax layer to align the second-order statistics between the center model and client models.

#### 5.1.2. Multi-Task Learning

Multi-task learning can be applied to solve multi-user adaptation and to mitigate the data heterogeneity issue across multiple users. Because focusing only on a single model may ignore the potential information in some related tasks that may promote the target task. By sharing parameters between different tasks to a certain extent, the original task may be better generalized to benefit all users.

Chen et al. [65] presented a multi-task learning method to solve multi-user data heterogeneity and improve the overall performance for all users simultaneously. This method aims to reduce person-specific discrepancy by aligning the marginal distributions of the labeled data and the unlabeled data and preserving task-specific consistency by generating paired data and maintaining consistency across their features. To achieve the goal, there are four tasks for learning synchronously: (1) the user adversarial loss that forces a reduction in the distribution divergence of the latent features of labeled and unlabeled data via Jensen–Shannon divergence; (2) the reconstruction loss that learns two decoders to reconstruct input vectors from latent features via an autoencoder; (3) the latent consistency loss, which is a constraint that avoids losing task-specific information during training; and (4) the final prediction loss that encourages the encoder to learn discriminative features and ensures a powerful label predictor is trained.

#### 5.1.3. Subject Clustering

Subject clustering highlights data sharing among similar subjects to form clusters to reduce sensor-based HAR data heterogeneity among all users. Therefore, the shared model in each cluster has a good generalization ability and can be applied to users without labeled data in the same cluster. Sztyler et al. [66] researched four cross-subjects approaches, namely Randomly, LOSO (Leave-One-Subject-Out), Top-Pairs, and Physical. Randomly means the data are chosen at random among subjects for training of the classifier, except the target user. The LOSO strategy is used to build a classifier for each subject that relies on all available labeled data except that of the target person. Top-Pairs involves comparing the subjects pairwise to find the best matches. Only in this case, new user labeled data are a requirement. In the Physical approach, the choice is made based on the idea that people with the same fitness level should have similar patterns and data distribution divergence should be small. So only users with similar fitness levels are clustered together. Then, all the labeled data in the same cluster are trained together to improve the general model performance and mitigate the data heterogeneity issue, even if some users have no labeled data. Similar to the above fitness-level clustering method, Ferrari et al. [67] explored the relationship between physical characteristics and signal similarity across different users. Similarity in physical characteristics such as age, weight, and height, as well as sensor signal similarity and the combination of these two types of sensitivity are under consideration.

#### 5.1.4. Batch Normalization

Batch normalization is a deep learning trick. It has attracted increasing attention with respect to solving the sensor-based HAR data heterogeneity issue because of its effectiveness in reducing covariate shift within deep neural network layers. Mazankiewicz et al. [68] added a domain alignment batch normalization layers to align the feature distributions of all subjects no matter whether they are from source domains or target domains in hidden neural network layers in order to reduce the negative effects of multi-user data heterogeneity. Domain alignment batch normalization reduces covariate shift across all users. Therefore, the deep neural network learns a feature transformation that maximizes class separability while making the domains overlap.

#### 5.1.5. Comparison of Methods

As shown in Table 7, federated learning was originally designed as a distributed machine learning method for shared data security and privacy. It is also a good user information sharing mechanism that can be applied to solve the issue of subject data heterogeneity. However, the generalized model may not work well for some specific users if the user data distribution is very different from the general model after the inappropriate selection of a federated learning strategy. Multi-task learning highlights the model generalization capability to solve the multi-user data heterogeneity issue via sharing of parameters between different tasks for the original task to be better generalized to benefit all users. However, how to elaborate multiple tasks to improve performance for all users still requires more effort. Subject clustering follows the unsupervised learning approach, with no requirement for labeled data. The key element is the appropriate selection of the clustering similarity metric, which is highly related to the task of reducing data heterogeneity cross users. Otherwise, inappropriate clustering metrics may cause a performance drop for all users. Batch normalization was originally designed as a deep neural network training trick. It is used to reduce covariate shift within deep neural network layers and. thus, to stabilize and accelerate training. Therefore, the usage of batch normalization for to handle the data heterogeneity issue has no extra computation cost. However, this method is a coarse-grained alignment for general data adaptation without considering the conditional distribution alignment. In other words, if two users’ classifiers’ decision boundaries are different, batch normalization does not work well. The corresponding human activity categories in each approach are also listed in Table 7. Federated learning covers all four types of activity categories. In contrast, multi-task learning is more commonly applied to handle atomic activity or composite activity. Subject clustering focuses on daily living activity and composite activity. Batch normalization considers daily living activity and sports fitness activity.

### 5.2. Single User Adaptation

Single user adaptation often refers to personalized model learning. Personalization means the process of capturing a user’s personal characteristics [11]. The dominant learning paradigm in this category is transfer learning, which transfers knowledge and aligns feature distribution from the source user(s) to the target user. In this way, sensor-based data heterogeneity between source user(s) and the target user can be mitigated, benefiting the model performance of the target user. Here, we divide transfer learning into more detailed categories, namely sample clustering, subspace mapping, deep neural networks, and ensemble learning.

#### 5.2.1. Samples Clustering

Some works have focused on sample clustering to assign the labels corresponding to the source user(s)’ samples to their neighboring samples from the target user. Fallahzadeh and Ghasemzadeh [69] proposed training a general model first from the source domains with labeled data. Once a new user emerges, the activity learner module extracts feature-based similarities between the source domain and target domain for the labeling of unseen data from the user. Finally, the personalized model is trained via using the labeled data. Here, clustering and weight bipartite matching are applied to unsupervised data annotation. Data are clustered first; then, the relationship between clusters and labels is learned by weight bipartite for label propagation.

Vo et al. [70] proposed a method to transfer a trained model from one user to another user via personalized adjustment. First, the labeled samples of person A are used to train an SVM classifier. Second, the model is directly used to classify the unlabeled samples of person B to obtain the data annotations. The unlabeled samples of person B are also clustered as the same number of the activity classes by the K-means and K-medoids algorithms. Third, only the confident samples are selected from the clustering, and the confident samples’ labels are the same as the cluster center points’ labels. The cluster center points’ labels are attained by model classification in the second step.

Zhao et al. [71] used a similar framework as Vo et al. [70] but considered the drawback of the K-means algorithm, where the clustering results and the number of iterations are dependent on the initial cluster centers and the iterative process can be very slow to converge with a bad initialization. Therefore, they added an extra restricted condition of a pre-collected labeled dataset with great similarity to the new user’s unclustered and unlabeled dataset. Then the initial center points with labels are set to be the cluster centers of this dataset before conducting K-means clustering steps on the unclustered dataset. After the clustering step, each sample is automatically labeled using the same annotation as the labeled initial point in each cluster. In contrast to common confident sample selection methods that ignore the effect of relative density on samples selection, they used a local outlier factor technique to remove some outliers with low relative density in each cluster. In the model classification training phase, a multivariate Gaussian distribution classifier is used because of its low computational complexity for wearable devices.

#### 5.2.2. Subspace Mapping

Subspace mapping is based on the assumption that there is a common subspace among different users and that the features in this subspace are invariant cross users. Therefore, finding the subspace and mapping different domains to the subspace can address the subject data heterogeneity issue.

Ye et al. [72] proposed a Temporal Relation Optimal Transport (TROT) subspace mapping method for single-user adaptation. This method utilizes both the hidden Markov model and optimal transport techniques to align temporal relations across source and target subjects. It achieves this by representing and aligning the temporal relations of activities, enhancing knowledge transfer between subjects. To further improve domain adaptation performance, TROT introduces a novel regularization term that preserves the order of temporal relations during optimal transport mapping.

Fu et al. [73] proposed a new transfer learning algorithm that combines improved pseudo-labels and the joint probability-domain adaptive method. The improved pseudo-label method uses supervised locality-preserving projection to learn the projection matrix to map the source domain and target domain to the same subspace. Only the source domain is used to obtain the projection matrix at the beginning, then assign pseudo-labels to the target domain. The nearest class prototype and structured prediction are applied to the label target domain. In contrast to common methods such as transfer component analysis and joint distribution adaptation that consider only either the marginal probabilities or conditional probabilities, the joint probability-domain adaptive method is derived from the inequality assumption of joint probabilities, which results in improved performance in between-domain transferability and between-class discrimination.

Liu et al. [74] focused on extracting constant information across users. For example, the duration of a healthy adult’s every single motion, such as jumping, sitting down, standing up, jogging (one gait), turning right, etc., clearly falls within a time length of about 1 to 2 s and is normally distributed among individuals. With relatively constant features cross users, the data heterogeneity issue can be mitigated. This paper analyzed the duration statistics and distributions of 22 fundamental individual movements of both everyday activities and sports activities. Moreover, in another work [75], human activity was partitioned into a sequence of distinguishing states for model generalization to find invariant information across users.

#### 5.2.3. Deep Neural Networks

A deep neural network has the capabilities of non-linear mapping and feature extraction. More and more works have focused on exploring deep neural network transfer learning for user adaptation.

Ding et al. [76] performed an empirical study on CNN-based deep transfer learning between users. Multiple-kernel maximum mean discrepancy, domain-adversarial neural networks and Wasserstein distance are three common CNN-based transfer learning components that are used to reduce the degree of sensor-based HAR data heterogeneity across domains. Moreover, center loss integrated into multiple-kernel maximum mean discrepancy loss is utilized to improve the cohesion of inner-class feature distribution among the source domain and target domain to further improve the adaptation performance of the target user model.

Some researchers have dropped the complete restriction of labeled data in the target domain, using a few labeled data that can cover all the activity classes for the new user. The fine-tuning method is the most popular method in deep neural network transfer learning when labeled target-domain data are available. This method assumes the lower layers of the neural network can extract common features among all users and that sensor-based HAR data heterogeneity only occurs in higher layers, which are specific to each user. Rokni et al. [77] introduced a CNN deep learning model for the fine tuning of cross-subject transfer learning. First, a deep network is trained by a group of users as the source domain. Then, the lower layers of the network are fixed, and the upper layers are re-trained with a few labeled data from the target user.

Emerging methods in deep neural networks increasingly leverage generative models to enhance performance. Compared to discriminative models, generative models offer several advantages: they can model the underlying data distribution, generate new samples, and better handle scenarios with limited labeled data by learning richer representations of the input space [78]. These characteristics make generative models particularly effective for tasks involving subject data heterogeneity. Ye et al. [79] introduced the Deep Generative Domain Adaptation with Temporal Attention (DGDATA) method. This innovative approach incorporates temporal dependency relations into the transfer learning process by leveraging generative models in conjunction with a temporal relation attention mechanism. This integration enhances classification performance in cross-user HAR tasks.

**Figure 7:**
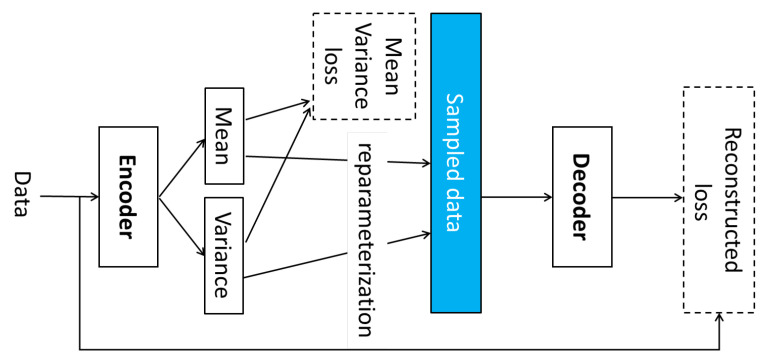
Variational autoencoder generative models.

Ye et al. [80] proposed the Conditional Variational Autoencoder with Universal Sequence Mapping (CVAE-USM) approach, a generative model designed to address challenges in cross-user activity recognition. This method combines the strengths of Variational Autoencoder (VAE) generative models as shown in Figure 7 and Universal Sequence Mapping (USM) to effectively capture and utilize common temporal patterns between users, significantly enhancing activity recognition performance. VAEs are probabilistic generative models that encode input data into a latent space and reconstruct them, enabling the modeling of complex distributions and the generation of realistic data samples. USM complements this by aligning and mapping sequences across domains to maintain consistency in temporal dynamics. Together, these components enable CVAE-USM to align user-specific activity patterns effectively, improving both generalization and accuracy in activity recognition tasks.

Another emerging area is the use of graph networks for domain adaptation. The SPA (Spectral Alignment) method [81] focuses on both inter-domain transferability and intra-domain structure. SPA transforms the domain adaptation problem by representing data as graphs, where nodes correspond to data points and edges capture their relationships (e.g., similarity or correlation). This method introduces a coarse graph alignment strategy enhanced by a spectral regularizer, which aligns the source- and target-domain graphs within a shared eigenspace. By leveraging the spectral properties of graphs, SPA minimizes domain discrepancies while preserving the intrinsic structure of each domain.

#### 5.2.4. Ensemble Learning

For user adaptation, ensemble learning provides a flexible adaptation approach for solving subject data heterogeneity. It can be used to train a weak classifier for the new user; then, a weight update is implemented to borrow previous knowledge from other weak classifiers. Casale et al. [82] trained a general classifier using an AdaBoost ensemble method with a large amount of data from many subjects. When a new user appears, the general classifier is applied to adjust its weight and adds the new weak classifier to its weak classifier set with the labeled data from the new user.

Hong et al. [83] trained a Bayesian network, a naïve Bayes classifier, and an SVM separately for each user’s activities to construct a pool of activity models. Each model is trained as a binary classifier with the labeled data. When a new user appears, the fitness of all models in the pool is measured with the new user’s small amount of labeled data. The models in the pool with high fitness values are selected to build a hybrid model for the new user. High fitness means low data heterogeneity between the selected model and the target user, and the fitness value is the weight of the hybrid model that shows how much of other users’ knowledge needs to be transferred to the new user.

#### 5.2.5. Comparison of Methods

As shown in Table 8, neither sample clustering or subspace mapping has a requirement for labeled target user data. However, sample clustering has the drawback of its high dependency on the sample clustering similarity metric. For subspace mapping, there is no ideal technique to select the optimal subspace dimension, which is important to achieve good performance for target user adaptation. The deep transfer learning method has a strong common feature extraction capability cross users considering the structure of deep neural networks. However, the computation cost is expensive, and model training requires more training tricks. Ensemble learning is a flexible adaptation approach for solving subject data heterogeneity. However, it needs to train an extra weak classifier every time a new user appears. The corresponding human activity categories in each approach are also listed in Table 8. Sample clustering, subspace mapping, deep neural networks, and ensemble learning all focus on daily living activity and sports fitness activity. In addition, sample clustering can also be applied to composite activity.

## 6. Spatial Data Heterogeneity

Sensor-based HAR heterogeneity in the spatial dimension includes two categories: environmental heterogeneity and body position heterogeneity. Environmental heterogeneity is associated with the fact that different environments have different sensor layouts. For example, we have the task of identifying the householders’ cooking activities in two smart houses with different sensor devices and/or sensor deployment positions. Environmental heterogeneity contains two types of data sources: ambient sensor data and device-free data. An ambient sensor is the traditional and most common data source that needs to be installed in advance for sensor reading and HAR. Device-free data represent a new research direction that focuses on analyzing wireless electromagnetic wave signal patterns for sensor-based HAR. In contrast, body position heterogeneity refers to cases where sensor data patterns can be different when the same sensor is deployed at different positions on a human body, such on an arm or leg. Different data patterns can happen even when people perform the same activity. For instance, the same sensor embedded in a shoe and in a smartwatch exhibits different data patterns when the user runs.

### 6.1. Environmental Heterogeneity

Many works have mainly focused on cross-subject comparisons in the same environment. Cross-subject and cross-sensor comparisons in different environments have been less explored. Sensor-based HAR data heterogeneity in an environmental layout refers to the different numbers of sensors, sensor deployment layouts, and other variations due to changing physical environments.

#### 6.1.1. Ambient Sensors

Smart homes are typically deployed with ambient sensors to identify human activities and describe environmental features. The most common method is to find a way to map the source sensor network to the target sensor network based on similarity. Essentially, the approaches addressing this issue typically discover the common latent characteristics and the feature relationship between the different physical environments. Therefore, the mainstream method is feature transformation under the transfer learning paradigm [15] across homes to solve sensor-based HAR spatial data heterogeneity caused by different physical environments. Most of the methods focus on feature extraction from temporal, spatial, and object interaction perspectives.

##### Transfer Learning

Chen et al. [84] combined four machine learning techniques for common feature discovery and feature transformation. PCA is used in the source house and target house separately to unify the heterogeneous features to the same dimensions in order to reduce data heterogeneity. Then, Jensen–Shannon divergence (JSD) is applied to measure the feature similarity between the source house and the target house. Lastly, the Gale–Shapley (GS) algorithm matches features based on the above similarity value between the source house and the target house for feature transformation.

Feuz and Cook [85] presented a method called feature-space remapping (FSR) to solve the spatial data heterogeneity issue. The FSR algorithm focuses on the challenge of the source and target domain coming from different feature spaces, which is very common in spatial data heterogeneity considering the different numbers of sensors and different sensor layouts deployed in different houses. Compared to traditional transfer learning, which involves a transfer from the source to target or a transfer from the source and target domains to a common feature space, FSR learns a mapping from each dimension in the target feature space to a corresponding dimension in the source feature space. FSR first selects and computes meta-features such as the average sensor event frequency over one hour and the mean and standard deviation of the time between sensor events. Then, a many-to-one similarity mapping matrix is generated between the source house and the target house by calculating each feature–feature pair based on meta-features. In addition to the basic FSR version, a genetic algorithm FSR and greedy search FSR [86] have been presented to further reduce data heterogeneity.

The above transfer learning methods are based on the data-driven approach. Some works have also focused on the knowledge-driven approach, especially ontology-based methods, which abstract activities as models of multiple entities, attributes, components, and relationships [87]. Because manual knowledge engineering conceptualizes and abstracts semantic and context processes for activity recognition, the knowledge-based method naturally has the capacity for generalization and dealing with spatial data heterogeneity. For example, in the heterogeneous deployment of sensors in homes, despite different physical layouts, the functional areas can be abstracted as, e.g., bedroom, kitchen, and toilet, which share similar characteristics.

Ye et al. [88] proposed a knowledge-driven method by introducing semantic relations in object sensor events. The ontological model includes four parts: object, location, sensor, and activity ontologies. Object and location ontologies are domain-independent because they typically occur in every household. For example, every house has a sleeping area and tableware. The sensor ontology and activity ontology depend on the specific application scenario—for instance, different sensor deployments based on the environment and different activities of interest. Next, the semantic similarity between sensor events is measured by temporal, spatial, and object features from the ontology model. After extracting all the unique sequences and mapping each activity, K-means clustering is applied to each activity sequence to discover the representative pattern of the activity based on semantic similarity. Lastly, sequential patterns are used in a new environment for activity recognition.

##### Multi-View Learning

Unlike the above works under the transfer learning paradigm, which has a transformation direction from the source to the target domain, Ye [89] extended multi-view learning to synchronously handle environmental layout heterogeneity across multiple houses. Here, each house can be treated as a view, and each view provides partial information. The assumptions are that each house can only have a small fraction of labeled data and that data from different houses are correlated in feature space. For each house, a classifier is trained, and an uncertainty measure is estimated using a small amount of labeled data. A set of unlabeled examples is generated by randomly selecting from all the houses’ unlabeled data. Each unlabeled example from the set is sent to its corresponding classifier to obtain its class probabilities. In addition, a confidence value is given to the unlabeled example via the uncertainty measure. Then, the most confident examples from the set with their predicted labels are used to complement the limited labeled data. A final classifier is trained based on the new and limited labeled data for all houses. In this way, spatial data heterogeneity is solved not in a house–house pair way but by learning a general model to cover the complete data distribution of all the houses’ sensor data.

##### Comparison of Methods

As shown in Table 9, the PCA + GS + JSD method requires less computational time because of the low computational complexity of this method. However, the feature mapping mechanism follows the one-on-one pairs method, which may cause information loss. For instance, sensor A is deployed in the bedroom of a small house and the bedroom is also used as an office area. Sensor B is deployed in the bedroom and sensor C is deployed in the office in a big house. In this case, sensor A’s data features should follow a one-to-many mapping that maps sensor B and sensor C.

FSR is a robust method that can solve environmental heterogeneity and feature mapping even if the heterogeneous feature spaces are in different houses, which is an ideal method considering the different sensor layouts in different houses. However, the FSR method highly depends on the choice of meta-features and may cause the performance of the target house model to drop dramatically in some scenarios. For instance, people have different daily routine habits; someone prefer to sleep early, while others tend to sleep late. In this case, the meta-features of average sensor event frequency over a certain time period may not be representative across different homes.

An ontology-based model is a knowledge-driven method that abstracts environments as models of object, location, sensor, and activity ontologies. Object and location ontologies are domain-independent because they typically occur in every household. In this way, environmental heterogeneity can be solved based on the definition of the same functional area. However, the model generation of location ontology may be inefficient if different house types. For example, the sleeping area and leisure area may overlap in a crowded urban house compared to a country house, which may lead to the inappropriate design of the location ontology.

In contrast to the above transfer learning paradigm, a sharing model follows the paradigm of multi-view learning, solving data heterogeneity a cross all houses at the same time. This method combines the information from all houses and benefits all houses as well. However, the success of this method is based on the assumption that different domains have the same set of activities. If there are no overlapped activities across houses, there is no useful information to share across the houses.

The corresponding human activity categories in the above four approaches are listed in Table 9. All four types of technique have been applied to composite activity, with exceptions for ontology-based and sharing model approaches, which can also be applied to daily living activity.

#### 6.1.2. Device-Free Activity Recognition

In recent years, device-free activity recognition has been an emerging research area that focuses on analyzing wireless electromagnetic wave signal patterns to classify different activities. Due to the physical phenomena of electromagnetic reflection, diffraction, and refraction, a person’s physical activities lead to changes in the propagation of surrounding wireless signals. However, wireless signals are sensitive and easily disturbed by the change of environments such as different house layouts or signal interference due to the operation of household appliances. Moreover, research on device-free based HAR data heterogeneity with respect to environmental layout is in early stages. The dominant technique in this field is deep learning-related transfer learning, which has the capabilities of automatic feature extraction, denoising, and complex model construction.

A deep transfer learning method [90] was applied to extract subject-independent and environment-independent features to solve the device-free environmental data heterogeneity issue. To eliminate the interference of specific environment and subject noise, a CNN-based deep domain discriminator is used to force the feature extractor to remove domain-dependent activity features. In addition, a confidence control constraint and smoothing constraint are proposed to handle the issues of overfitting and fluctuating latent space, with a balance constraint confining the percentage of each activity in the final prediction to be the same as the percentage of prior knowledge. The highlight of the research is the evaluation and comparison of numerous device-free data types, including ultrasound, mmWave, visible light, and Wi-Fi signals.

With a similar idea of finding domain-independent features, Zheng et al. [91] proposed a deep transfer learning method and constructed a cross-domain gesture recognition feature called the Body-coordinate Velocity Profile (BVP) under the assumption of different locations and orientations relative to Wi-Fi links and environments. Time-frequency analysis and motion tracking originating from channel state information (CSI) signals are used to generate the Doppler frequency shift profile, as well as orientation and location information. Then, the BVP feature is refined from this information by compressed sensing BVP estimation. Lastly, a GRU + CNN deep learning one-fits-all model is applied to extract temporal and spatial features for gesture recognition. In addition, a Wi-Fi gesture recognition dataset called Widar3.0 has been released to the research community.

Wang et al. [92] developed a CNN-based deep transfer learning model to transfer target-domain feature distributions to the source-domain distribution. The ingenious design of the method lies in the process of maximizing the decision discrepancy of the classifier to tightly encircle the feature distribution through classifier model adjustment. An adversarial technique was developed to guide the feature extractor using a CNN-based deep neural network to move the target features to the distribution of the source features. Then, an alignment technique is applied to relocate the target features to the distribution center of the source domain to further attain a uniform distribution of the target domain.

Compared to the above methods using spatial and temporal feature extraction directly without particular intention, Shi et al. [93] aimed to enhance activity-dependent features and ignore the activity-unrelated features such as signal reflection from the background static objects. The raw CSI signal contains multiple channel paths from static background objects and, hence, a lot of activity-unrelated information. Such information is generally environment-dependent, and the spatial data heterogeneity issue is obvious. To solve the data heterogeneity issue, the authors used a linear recursive operation to construct the CSI signal for static objects, then subtract it from the received signal to obtain activity-unrelated information. Then, they extracted useful features from the filtered CSI, including two pieces of activity-related information and a temporal correlation feature for the following activity recognition task.

As shown in Table 10, the domain discriminator is efficient if two houses have and overall data heterogeneity issue and the decision boundaries of the classifiers should be similar to each other, which means it works well in marginal distribution alignment. However, the domain discriminator is only applied at the domain level, which is coarse-grained alignment without considering the conditional distribution alignment. Therefore, if the decision boundaries of the classifiers are very different, this method fails to work. BVP is a newly proposed feature presentation that is domain-independent across houses. Therefore, with this common feature, the environmental heterogeneity issue can be solved. However, the derivation of this feature depends on some signal processing techniques. The uncertainty of the number of wireless links required to uniquely recover the BVP in different environments may cause the computational complexity to be hard to estimate in advance. The adversarial method does not require labeled samples in the target domain, which has a wider application scenario. However, the step of maximizing the classifier’s decision discrepancy may lead to a model overfitting problem and affect the transfer learning performance. The activity-unrelated information filter method has a good generalization capability, regardless of environmental change. However, it needs an extra step to generate a CSI signal pattern for each new environment. The corresponding human activity categories in each approach are also listed in Table 10. BVP and adversarial methods focus on atomic activity. A domain discriminator considers daily living activity and composite activity. An activity-unrelated information filter aims to handle atomic activity, composite activity, and daily living activity.

### 6.2. Body Position Heterogeneity

Wearable device and smartphone activity recognition commonly assumes a fixed sensor position on the body to simplify the operation. However, this assumption is sometimes difficult to hold in practical applications because the physiological structure of the human body leads to very different distributions of sensor data from various body parts, even with the same activity. For example, when people are running, the acceleration signal fluctuates intensely in the ankle compared to the waist [94]. There are usually two main methods in this area: (1) position-aware methods that take into consideration the sensor’s position and (2) position-independent methods that make use of some on-body position-independent features.

#### 6.2.1. Position-Aware Method

Compared to the sensor-based HAR data heterogeneity issue in different subjects, the issue of sensor-based HAR data across body positions is relatively more manageable because of the limited number of human positions such as waist, ankle, arm, and leg positions. An enumeration method with standard supervised learning is possible in this field, which means the test dataset belongs to one of the training datasets. Transfer learning is the other learning paradigm that transfers knowledge from one or more body position(s) to the target body position.

##### Enumeration Method

Yang et al. [95] also identified smartphone sensor positions using a decision tree model. After adjusting the accelerometer data corresponding to the position, an SVM is trained for activity classification. To eliminate the influence of smartphone orientation, the coordinate system is converted from triaxial acceleration directions to vertical and horizontal directions.

Sztyler et al. [96] considered the influence of different on-body positions of a mobile device and predicted the sensor position based on acceleration data via a random forest classifier. The stratified activity recognition method includes dynamic behavior (climbing, jumping, running, and walking) and static behavior (standing, sitting, and lying) with the features of time (such as entropy, correlation coefficient, and kurtosis) and frequency (such as Fourier transformation).

##### Transfer Learning

Transfer learning is another learning approach in this area. Wang et al. [9] presented a transfer learning method for the position-aware body position heterogeneity issue. The idea of the framework includes three steps of majority voting to generate pseudo-labels for the target body position via classifiers trained on source body positions, intra-class transfer into the same subspace between source body positions and target body positions via maximum mean discrepancy similarity calculation, and a second annotation in the target domain. The second and third steps are the iterative refinement of the Expectation-Maximization (EM) algorithm until convergence. As an extension of the work reported in [9], Chen et al. [97] further added a stratified domain selection component that can select the source body position the most similar to the target body position. It is a greedy algorithm that exploits the local properties of multiple source body positions via the Radial Basis Function (RBF) kernel method.

Similar to Chen et al.’s algorithm for choosing the right source domain for better performance and prevention of the negative transfer, Wang et al. [98] proposed source body position selection with a different distance measurement method called context activity distance. It is an overall distance including kinetic distance for approximation of the signal relationship between source body positions and target body position, using semantic distance to indicate the spatial relationship between the source domains and target domain. Deep learning is applied to transfer knowledge and activity recognition, and maximum mean discrepancy is the last step used to reduce the discrepancy between domains for the adaptation layer.

Rokni et al. [99] proposed a transfer learning method to meet the requirement of model adjustment in real time when new sensors are deployed at different body positions without labeled data. The existing sensors are treated as source domains, and the emerging sensors are treated as the target domain. Source-domain data are first utilized to generate a likelihood function of existing sensors’ activity classification by calculating the occurrence rate of each pair of the predicted label and actual label at a certain time point. Simultaneously, target data use this likelihood function as their classifier and are combined with the source data to form a new dataset. Clustering is implemented on this new dataset, and the number of cluster groups is the same as the number of activity classes. Afterwards, a mapping function is learned to link the weight relationship between the clusters and activity classes. Finally, the best matched class is assigned to the corresponding cluster and the instances in it for autonomous annotation.

##### Comparison of Methods

As shown in Table 11, the enumeration method achieves high accuracy in identifying body positions. This method does not focus on reducing body position heterogeneity but builds a model for each body position separately. However, the main cost is associated the requirement for labeled data from all body positions that are rare in the real world. Transfer learning has no requirement for labeled target body position data. However, the success of solving body position heterogeneity depends on the cross-domain similarity metric, which is suitable for reducing body position heterogeneity. The corresponding human activity categories in each approach are also listed in Table 11. Both the enumeration method and transfer learning focus on daily living activity and sports fitness activity. In addition, transfer learning can also be applied to address composite activity.

#### 6.2.2. Position-Independent Method

The dominant method here is multi-view learning, which finds some insensitive features via mixed data from all available body positions to achieve model generalization capacity. Zhou et al. [100] proposed a hierarchical method that distinguishes vertical-direction, coarse-grained activities in the first layer, then identifies fine-grained activities in the second layer. A barometer sensor is used to identify altitude changes for the classification of horizontal walking up and down stairs. After feature extraction through wavelet transform and singular value decomposition, machine learning models identify six types of upstairs and downstairs actions with different movement velocities. The models then recognize five fine-grained walking modes and generate domain-independent features without considering the phone position. Almaslukh et al. [101] also combined the data from all positions to train a general model. In addition, they performed comprehensive research on sensor-based HAR data heterogeneity in body position.

Unlike the above method treating each body position as a view for multi-view learning, Wang et al. [94] treated each sensor as a view for multi-view learning and applied sensor fusion to generate magnitude series to eliminate the orientation information, then extracted statistical and frequency-domain features. After PCA dimension reduction, the obtained principal components are utilized to train the classification model via a mixed kernel-based extreme learning machine algorithm. Afterwards, the most confident samples, combined with initial training data, are selected to update the ELM classification model. The renewed model gradually adapts to data in previously unseen locations as the emerging new data from a new sensor location.

## 7. General Framework for Multiple Heterogeneities

Some researchers presented a general framework to handle multiple sensor-based HAR data heterogeneity issues with a sufficient and complete experiment other than the four categories of sensor-based HAR data heterogeneity issues discussed in the previous sections: data modality heterogeneity, streaming data heterogeneity, subject data heterogeneity, and spatial data heterogeneity. Some research has focused on solving sensor-based HAR mixed data heterogeneity scenarios. Here, the transfer learning paradigm is the dominant method.

To better align source-domain and target-domain features, some methods require source-domain data and labeled target-domain data. Feng et al. [102] proposed a general cross-domain deep transfer learning framework combined with parameter-based transfer learning and cross-domain class-wise similarity. In the first step, a stacked LSTM network with a feature extractor and a classifier is trained, and the training data are source-domain samples. In the second step, the network parameters of the source feature extractor and classifier are transferred to a target network with the same structure. In particular, the weight of the transferred source classifier parameters is dependent on a cross-domain class-wise relevance measure, which includes cosine similarity, sparse reconstruction, and semantically normalized Google distance. Then, the transferred classifier parameters and the source feature extractor parameters are used to initialize the target network. In the last step, fine tuning is applied to adjust the feature extractor and classifier in the target network.

Some work only requires labeled source-domain data and unlabeled target-domain data. Sanabria et al. [103] proposed a transfer learning framework from the source domain(s) to the target domain via Bi-GAN. The architecture of Bi-GAN consists of two GANs of a generator and a discriminator in the source and target domain, respectively. In the feature-space transformation step, both generators are trained to generate fake samples as close to the real samples in the other domains as possible, and both discriminators are binary classifiers to detect whether an input is generated by their corresponding generators or a real sample from the other domains. In the feature distribution alignment step, the transformed features are shifted to the real target data via kernel mean matching to improve classification accuracy. Then, a classifier is trained on the aligned transformed features, and the features’ corresponding labels are inherited from the source domain. Finally, the classifier is used to label data in the target domain.

Domain generalization is an emerging area of transfer learning that explores how to acquire knowledge from various related domains. It only assumes that samples from multiple source domains can be accessed and target domain can provide no data at all. Limited work has been done in this field. Erfani et al. [104] proposed the elliptical summary randomization framework for domain generalization comprising a randomized kernel and elliptical data summarization. The data are projected to a lower-dimensional latent space using a randomized kernel, which reduces domain bias while maintaining the data’s functional relationship. The Johnson–Lindenstrauss method provides probabilistic assurances that a dataset’s relative distances between data points are preserved when randomly projected to a lower feature space. Therefore, ellipsoidal summaries are employed to replace the samples to increase generalization by reducing noise and outliers in the projected data. The focal distance between pairs of ellipsoids is then used as a measure of domain dissimilarity. Lu et al. [105] considered global correlation and local correlation of time-series data. Local and global features-learning and alignment framework was proposed for generalizable human activity recognition.

## 8. Public Datasets

### 8.1. General Datasets

There are some public datasets that can be applied to evaluate various types of sensor-based HAR data heterogeneity issues. Here, we summarized the datasets that are used in two or more fields of heterogeneity issues. These eight datasets may attract more attention because of their versatility and ability to conduct different types of evaluation under various scenarios.

Table 12 shows the corresponding research and evaluation areas of specific data heterogeneity issues to which each dataset can be applied. The numbers in brackets indicate the number of papers that have used this dataset in the specific field. The key information of these eight datasets is also summarized in Table 13.

### 8.2. Specific Datasets

There are some public datasets designed for a specific and particular sensor-based HAR heterogeneity problem. Here, we summarize the specific datasets with more two or more cited papers. These eleven datasets may also be considered when researchers want to dive into a specific sensor-based HAR data heterogeneity problem.

Table 14 shows the specific data heterogeneity fields and their corresponding problem field-dependent datasets. The key information of these eleven datasets is also summarized in Table 15.

## 9. Future Directions

The research work surveyed in this review also identified some future research directions that require further discussion.

**Figure 8:**
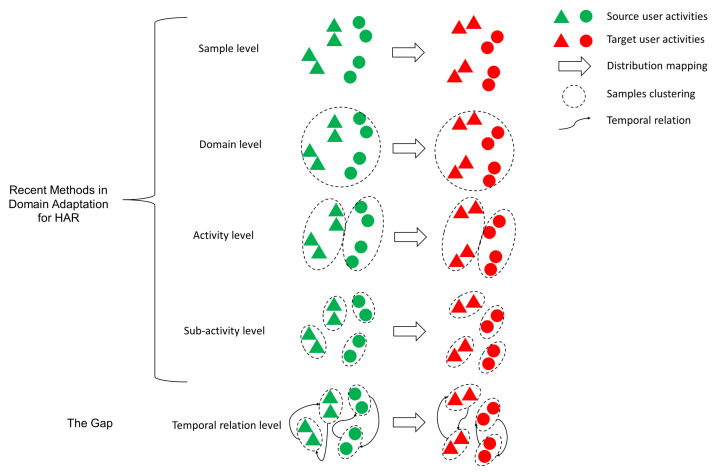
Different levels of domain adaptation.

Temporal relationship integration in domain adaptation is a critical and emerging area that requires focused attention in sensor-based HAR. Temporal dynamics are fundamental to HAR, as they capture the sequential dependencies and interactions within time-series data. Despite their importance, current methods often neglect temporal relationships or address them in a limited manner, leading to suboptimal performance when transferring knowledge across domains, as shown in Figure 8. Recent methods primarily focus on sample-level, domain-level, activity-level, and sub-activity-level domain adaptation for data distribution mapping, whereas temporal relation-level mapping remains less explored. Therefore, future research should prioritize the development of domain adaptation techniques that explicitly model and incorporate temporal relationships to enhance alignment between source and target domains. Methods leveraging temporal patterns can significantly improve generalization, particularly in scenarios involving cross-user or cross-device data heterogeneity. Exploring advanced temporal modeling approaches such as temporal graph networks or attention-based mechanisms could pave the way for breakthroughs in this area.

Cross-modality knowledge transformation in sensor-based HAR is a new and promising research direction. There is still very limited work in this area. Its purpose is to build a bridge and find the relationships between different data modalities to take advantage of knowledge from the source domain, especially under scenarios of difficult data collection from the target domain. If the gap between some modalities’ feature spaces is relatively big and these domains have no direct correlations, it may be difficult to achieve cross-modality knowledge transformation via traditional transfer learning methods. Transitive transfer learning (TTL) [123] is a potential solution inspired by the idea of transmissibility for indirect reasoning and learning. This method has the capability of assisting in connecting concepts and passing knowledge between seemingly unrelated concepts. Normally, the bridge is built to link unrelated concepts by introducing intermediate concepts. TTL is an important extension of traditional transfer learning to enable knowledge transformation across domains with huge differences in data distribution. There are two key points the need to be considered: (1) how to select the appropriate intermediate domain as the bridge between the source domain and target domain and (2) how to transfer knowledge efficiently across the connected domains.

The use of adaptive methods for unseen activities is another relatively new topic in sensor-based HAR at present and can be applied to more challenging and practical scenarios. It aims to learn a model that can generalize to a target domain with one or several differences but related source domain(s). It only assumes that samples from multiple source domains can be accessed and has no access to the target domain. For HAR, this research topic is suitable for various situations. For example, the activity data originate from multiple existing users (source domains) are difficult to obtain from a new user (target domain). In this case, the methods of domain generalization can be explored to handle this type of challenge. Moreover, extra semantic information can be introduced as external Supplementary Information. Current research focuses on embedding word vectors such as Word2Vec that build the semantic connections between the activity verbs and object nouns to transfer the classification label space to word vector space. Other types of external Supplementary Informationmay also be explored and introduced to help adaptive methods for unseen activities. For example, research on the the human skeleton and muscle structure in the medical field can be applied to HAR.

## 10. Conclusions

Human activity recognition (HAR) is an essential part of ubiquitous computing that aims to identify and analyze human behavior from multi-dimensional observations and contextual information. Its many application scenarios include medical treatment, auxiliary environment life, health, fitness and sports monitoring, rehabilitation, home automation, security monitoring, etc. Despite the tremendous research efforts made in the past few decades, there are still many challenges and emerging aspects of activity identification. Most of the latest generation of HAR methods are performed under the assumption of data homogeneity. Data homogeneity means that the data distribution is the same in all datasets. However, actual human activity sensor data are often heterogeneous. Solving sensor-based HAR data heterogeneity leads to improved performance with lower computational cost and helps to build a robust, adaptable, and custom model with less annotation work required. With the recent research development in sensor-based HAR data heterogeneity, this paper analyzed research works and discovered areas that still require further investigation. This review paper categorized different types of data heterogeneity in sensor-based HAR applications. Different machine learning techniques developed for each type of data heterogeneity were analyzed, compared, and summarized, along with their advantages and disadvantages. The available public datasets were also summarized to facilitate future research on sensor-based HAR data heterogeneity.

## Figures and Tables

**Table 1:** Categories of sensor-based human activity.

Activity Category	Activities
Atomic activity	Right arm throw, two hands front clap, frontal elevation of arms, waist bends forward, kicking, pocket out, flexion of the leg up, knees bending, draw zigzag, push, pull, slide, stand up, sit down, etc.
Daily living activity	Brush teeth, talking on phone, cleaning, eating walking, sitting, standing, lying, etc.
Sports fitness activity	Running, skiing, jogging, biking, playing basketball, exercising on stepper, rowing, rope jumping, dumbbell curl, squat upright row, Nordic walking, flick kicks, climbing, etc.
Composite activity	Make coffee, groom, cooking, clean counter tops, bathe, preparing breakfast, empty room, attend a presentation, housekeeping, using the toilet, personal hygiene, open/close door, etc.

**Table 2:** Sensor types, names, and corresponding activity categories in HAR.

Sensor Type	Sensor Name	Activity Categories
Inertial Measurement Unit (IMU) Sensors	Accelerometer, Gyroscope, Magnetometer	Atomic activity, Daily living activity, Sports fitness activity, Composite activity
Ambient Sensors	Pressure/Force, Infrared, Magnetic Switches/Contact, Ultrasonic, Light, Temperature, Humidity	Daily living activity, Composite activity
Device-free Sensors	RFID, Wi-Fi, Radar	Atomic activity, Daily living activity

**Table 3:** Comparison of modality fusion methods in sensor-based HAR.

Fusion Strategy	Techniques	Pros	Cons	Activity Categories
Data-level/Feature-level Fusion	Concatenation [4,30], self-attention [31], and clustering [32]	Considers the weighted contributions of all modality features/data	The need to unify different modality features/data into the same format	Atomic activity, daily living activity, and sports fitness activity
Classifier-level Fusion	Stacking ensemble [33], voting strategy [34], and attention weighting [35]	Decouples the features in each modality, making model training convenient	The need to deal with inconsistent results across classifiers	Daily living activity

**Table 4:** Comparison of modality fusion methods in sensor-based HAR.

Method	Data Modality	Labelled Data Requirement	Pros	Cons	Activity Categories
Fine tuning [36]	Video camera, audio, and wearable sensors	Source domain + target domain	More efficient than training a model from scratch	Fine Tuning may not be effective if a new task is very different from the original one	Daily living activity and sports fitness activity
Multi-view Transfer Learning [37]	Ambient sensors, smartphone/wearable sensors, and video cameras	source domain (+target domain)	Can be applied to scenarios without labeled target-domain data	More views make the training more computationally expensive	Composite activity
Knowledge Distillation [38]	Wearable sensors and video cameras	Source domain + target domain	The distilled model may generalize better to new data	Loss of information may occur	Daily living activity and atomic activity
Generative Network [40]	Video cameras and accelerometers	Source domain + target domain	Can produce a large amount of synthetic data	The quality of generated data may be lower than that of real data	Daily living activity and sports fitness activity

**Table 5:** Comparison of concept drift methods in sensor-based HAR.

Methods	New Labeled Data	Feedback Level	Pros	Cons	Activity Categories
Adaptive Activity Recognition Framework [42]	Yes	High	Compatibility and scalability	The need for more human and outside guidance	Sports fitness activity
Cluster + Ensemble Classifier [43]	Yes	Median	Can rectify the model effectively	The need for some human feedback	Daily living activity and sports fitness activity
Symbolic Data Discretization + Frequency Distributions [44]	No	Low	Fewer computational resources required	Sensor value compression uncertainty	Daily living activity
Knowledge-based [46]	No	Low	Does not require training data	Nay not be flexible enough and lead to false detection	Composite activity

**Table 6:** Comparison of Concept evolution learning paradigms in sensor-based HAR.

Learning Paradigm	New Labeled Data	Pros	Cons	Activity Categories
Zero-shot Learning	No	No need for new labeled concepts	Performance may drop dramatically due to weak relations between old concepts and new concepts	Daily living activity, sports fitness activity, atomic activity, and composite activity
Few-shot Learning	Yes	Able to learn new concepts	Incomplete data distribution	Daily living activity, sports fitness activity, and atomic activity

**Table 7:** Comparison of multi-user adaptation methods in sensor-based HAR.

Technique	Pros	Cons	Activity Categories
Federated Learning	Good user information sharing mechanism	Generalized model may not work well for a specific user	Daily living activity, sports fitness activity, atomic activity, and composite activity
Multi-task Learning	Good model generalization capability	Extra efforts required for multiple elaborated tasks	Atomic activity nd composite activity
Subject Clustering	No need for labeled data	Inappropriate selection of clustering similarity metric may reduce performance	Daily living activity and composite activity
Batch Normalization	No extra computation cost	Not suitable for conditional distribution alignment	Daily living activity and sports fitness activity

**Table 8:** Comparison of single-user adaptation methods in sensor-based HAR.

Technique	Pros	Cons	Activity Categories
Sample Clustering	No need for labeled target user data	Highly dependent on the sample clustering similarity metric	Daily living activity, sports fitness activity, and composite activity
Subspace Mapping	No need for labeled target user data	The optimal subspace dimension is hard to decide	Daily living activity and sports fitness activity
Deep Neural Networks	strong common feature extraction capability across users	Computationally expensive	Daily living activity and sports fitness activity
Ensemble Learning	Flexible adaptation with updated weights	Need to train a new weak classifier every time a new user appears	Daily living activity and sports fitness activity

**Table 9:** Comparison of methods addressing environmental heterogeneity in sensor-based HAR using ambient sensors.

Technique	Learning Paradigm	Learning Approach	Pros	Cons	Activity Categories
PCA + GS + JSD [84]	Transfer Learning	Data-driven	Less computation time	One-on-one feature mapping may cause information loss	Composite activity
FSR [85,86]	Transfer Learning	Data-driven	Can relate features in heterogeneous feature spaces	Highly dependent on the suitable choice of meta-features	Composite activity
Ontology-based Model [88]	Transfer Learning	Knowledge-driven	Good model generalization	Location ontology may be inefficient in different house types	Composite activity and daily living activity
Sharing Model [89]	Multi-view Learning	Data-Solves data heterogeneity across all houses	Does not work if different domains have different sets of activities	Composite activity and daily living activity	

**Table 10:** Comparison of methods addressing environmental heterogeneity in sensor-based HAR using device-free sensors.

Deep Transfer Learning Technique	New Labeled Data	Pros	Cons	Activity Categories
Domain Discriminator [90]	No	Good at marginal distribution alignment	Coarse-grained alignment without considering the conditional distribution alignment	Daily living activity and composite activity
BVP [91]	No	The extracted features are domain-independent	Uncertainty of the number of wireless links required to uniquely recover the BVP	Atomic activity
Adversarial Method [92]	No	No need for labeled samples in the target house	Nay cause overfitting after the step of maximizing the classifier’s decision discrepancy	Atomic activity
Activity-unrelated Information Filter [93]	Yes	Good generalization, regardless of environmental change	Need to generate a CSI signal pattern for each new environment	Atomic activity, composite activity, and daily living activity

**Table 11:** Comparison of position-aware methods for addressing body position heterogeneity.

Technique	Pros	Cons	Activity Categories
Enumeration Method	High accuracy in identifying body positions	Requires labeled data from all body positions	Daily living activity and sports fitness activity
Transfer Learning	No need for labeled target body position data	performance depends on the cross-domain similarity metric	Daily living activity, sports fitness activity, and composite activity

**Table 12:** General datasets and types of data heterogeneity.

Dataset and Ref.	Data Modality Heterogeneity	Concept Drift (Streaming Data Heterogeneity)	Concept Evolution (Streaming Data Heterogeneity)	Open-Set (Streaming Data Heterogeneity)	Subject Data Heterogeneity	Body Position Heterogeneity (Spatial Data Heterogeneity)	Multiple Heterogeneities
HHAR [32]	✓(3)	✓(2)			✓(2)		✓(3)
MHEALTH [106]	✓(2)				✓(1)	✓(1)	
OPPT [107]	✓(1)	✓(2)	✓(4)	✓(1)	✓(1)	✓(2)	✓(6)
WISDM [108]		✓(2)			✓(2)		✓(1)
UCIHAR [109]		✓(1)	✓(1)		✓(5)		✓(3)
PAMAP2 [110]			✓(5)		✓(4)	✓(2)	✓(6)
DSADS [111]			✓(1)		✓(5)	✓(2)	✓(4)
RealWorld [96]					✓(3)	✓(2)	

**Table 13:** Comparison of general datasets on sensor modalities, number of sensors, number of participants, and number and types of activities.

Dataset and Ref.	Sensor Modalities	No. of Sensors	No. of Participants	No. of Activities	Activity Categories
HHAR [32]	Accelerometer and gyroscope	36	9	6	Daily living activity and sports fitness activity
MHEALTH [106]	Accelerometer, gyroscope, magnetometer, and electrocardiogram	3	10	12	Atomic activity, daily living activity, and sports fitness activity
OPPT [107]	Acceleration, rate of turn, magnetic field, and reed switches	40	4	17	Daily living activity and composite activity
WISDM [108]	Accelerometer and gyroscope	1	33	6	Daily living activity and sports fitness activity
UCIHAR [109]	Accelerometer and gyroscope	1	30	6	Daily living activity
PAMAP2 [110]	Accelerometer, gyroscope, magnetometer, and temperature sensor	4	9	18	Daily living activity, sports fitness activity, and composite activity
DSADS [111]	Accelerometer, gyroscope, and magnetometer	45	8	19	Daily living activity and sports fitness activity
RealWorld [96]	Acceleration	7	15	8	Daily living activity and sports fitness activity

**Table 14:** Specific datasets and types of data heterogeneity.

Data Modality	Concept Evolution	Different Subjects	Environment Layout	Multiple Heterogeneities
UTD-MHAD [112]	Exercise Activity [113]	USC-HAD [114]	van Kasteren [115]	Skoda [116]
	TUD [117]	Shoaib [118]	CASAS [119]	
		SHAR [120]	Widar3.0 [91]	
		MobiAct [121]		
		Motion Sense [122]		

**Table 15:** Comparison of specific datasets on sensor modalities, number of sensors, number of participants, and number and types of activities.

Dataset and Ref.	Sensor Modalities	No. of Sensors	No. of Participants	No. of Activities	Activity Categories
Exercise Activity [113]	Accelerometer and gyroscope	3	20	10	Sports fitness activity
UTD-MHAD [112]	Accelerometer, gyroscope, RGB camera, and depth camera	3	8	27	Atomic activity, daily living activity, sports fitness activity, and composite activity
Shoaib [118]	Accelerometer and yroscope	5	10	7	Daily living activity and sports fitness activity
TUD [117]	Accelerometer	2	1	34	Daily living activity, sports fitness activity, and composite activity
SHAR [120]	Accelerometer	2	30	17	Atomic activity, daily living activity, and sports fitness activity
USC-HAD [114]	Accelerometer and gyroscope	1	14	12	Daily living activity and sports fitness activity
MobiAct [121]	Accelerometer, gyroscope, and orientation sensors	1	50	13	Atomic activity and daily living activity
Motion Sense [122]	Accelerometer and gyroscope	1	24	6	Daily living activity
van Kasteren [115]	Switches, contacts, and passive infrared (PIR)	14 (23) (21)	1 (1) (1)	10 (13) (16)	Daily living activity and composite activity
CASAS [119]	Temperature and infrared motion/light sensors	52 (63)	1 (1)	7 (9)	Daily living activity and composite activity
Skoda [116]	Accelerometer	19	1	10	Daily living activity and composite activity
Widar3.0 [91]	Wi-Fi	7	1	6	Atomic activity
